# The Pivotal Role of the Key Angiogenic Factors in the Development of Endometrioid Pathologies of the Uterus and Ovary

**DOI:** 10.3390/cancers16162772

**Published:** 2024-08-06

**Authors:** Gabriela Sabolová, Ivana Špaková, Peter Artimovič, Peter Bohuš, Miroslava Rabajdová, Mária Mareková

**Affiliations:** 1 Department of Medical and Clinical Biochemistry, P. J. Šafárik University in Košice, Trieda SNP 1, SK-04011 Košice, Slovakia; gabriela.sabolova@student.upjs.sk (G.S.); peter.artimovic@student.upjs.sk (P.A.); miroslava.rabajdova@upjs.sk (M.R.); maria.marekova@upjs.sk (M.M.); 2 Department of Pathology, P. J. Šafárik University in Košice, Trieda SNP 1, SK-04011 Košice, Slovakia; peter.bohus@student.upjs.sk

**Keywords:** angiogenesis, VEGF-A, TGF-β1, ANG1, ANG2, HIF-1α, endometriosis, endometrioid adenocarcinoma, endometrial carcinoma

## Abstract

**Simple Summary:**

Angiogenesis is a synchronous mechanism of new blood vessel formation that is controlled by pro- and anti-angiogenic factors. Angiogenesis differs from tissue to tissue, especially in pathological conditions. The exact nature of the co-operation between angiogenic markers is still unknown. Herein, we describe the possible critical changes in VEGF-A, TGF-β1, ANG1, ANG2, and HIF-1α in ectopic endometriosis (with elevated pro-angiogenic factors in all studied fields), in ovarian endometrioid adenocarcinoma (showing typical upregulation on the TGF-β1 axis), and two different types of endometrial carcinoma (with an extreme pathological ANG2/ANG1 axis for mixed mesodermal tumors). Understanding the angiogenic mechanism within pathological tissues could help to improve angiogenic treatment strategies and lead to better prognoses.

**Abstract:**

A characteristic feature of uterine pathologies is a specific change in cell metabolism, which predominantly manifests as a shift in the need for nutrients, thereby directing cells to engage in different angiogenic marker activities. Angiogenesis is one of the main signals supporting the survival and development of cells and tissues not only under physiological conditions. Therefore, it is necessary that we understand pathological hyperactivation in all uterine diseases, from endometriosis through ovarian endometrioid adenocarcinoma to malignant transformed cells of the uterine epithelium and body. This work presents the gene expression results of selected angiogenesis targets (VEGF-A, TGF-β1, ANG1/2, and HIF-1α), cell migration, and cell–cell interaction determined in vitro. Our results suggest that angiogenesis varies in the tested pathological conditions (ectopic endometriosis—12Z; ovarian endometrioid adenocarcinoma—A2780; tumors—SK-UT-1 and RL-95-2) compared to physiological angiogenesis (HME1). The differential expression of angiogenic factors may contribute (or is a contributing factor) to the observed differences to acknowledge an inherent variability in angiogenesis among cell lines. Determining the genomic phenomena responsible for processes associated with inadequate angiogenesis in the pelvic region could help us to develop individual treatment strategies and explain resistance to treatment.

## 1. Introduction

Physiological and properly regulated angiogenesis, the process of new blood vessel formation, is essential for the normal function of the reproductive system and has an important role in follicular maturation, corpus luteum development, and endometrial growth [1]. Dysregulated angiogenesis (or excessive growth of new blood vessels) can contribute to the development and progression of gynecological diseases such as the growth of ectopic endometrial tissue in endometriosis; tumor growth in the uterine cavity or ovaries; and the spread of disease into surrounding tissues. Endometriotic lesions and solid tumors can induce angiogenesis, which is a fundamental aspect of their survival because it ensures a constant diffusional exchange of nutrients, metabolites, and oxygen [2]. Establishing a solid blood supply allows the pathological tissue to persist and grow. A crucial aspect of angiogenesis is the sophisticated biochemical interplay engaged in by seven forms of vascular endothelial growth factor (VEGF-A/B/C/D/E/F and placental growth factor) [3]; as a central master regulator of angiogenic processes in the uterine cavity, it controls the entire angiogenic process [4]. The effect of VEGF transcription factors is mediated by their receptors, VEGFR1 and VEGFR2, which act through the tyrosine kinase pathway (also involved in angiogenesis); it is also mediated by VEGFR3, which is engaged in lymphangiogenesis [5]. VEGF-A (often referred to as VEGF), which has an anti-apoptotic effect on endothelial cells, stimulates proliferation, permeability, migration, and capillary tube assembly [6]. Previous studies in mice and rabbits have shown that VEGF and its receptors upregulate angiogenesis and control the vascular permeability required for implantation [7]. The effect of VEGF is mediated by its binding to its two tyrosine kinase receptors: VEGFR-1/Flt-1 (Fms-like tyrosine kinase-1), to which it binds with high affinity, and VEGFR-2/KDR (kinase insert domain receptor), which mediates most of the biological effects of VEGF [8]. The VEGF-A/VEGFR-2 signaling pathway plays a key role, as VEGF-A or VEGFR-2 deficiency leads to early lethality due to abnormal vascular development. The role of VEGF in the rapid onset of endometrial angiogenesis that occurs during postmenstrual regeneration and in the early proliferative phases results from hypoxia in endometrial stromal cells [6]. In addition, VEGF-A is oversynthesized in epithelial, mesenchymal, and tumor cells [9]. However, VEGFRs are expressed not only in vascular endothelial cells but also in macrophages and monocytes [5], which suggests that they have a role in immune responses. 

Hypoxia-inducible factor 1-alpha (HIF-1α) is emerging as another critical component of cell survival and adaptive response function. In a hypoxic environment, angiogenic factors bind to their receptor, which is present on the surface of endothelial cells, thereby promoting their dilation and activation [10]. The HIF-1α transcription factor is a molecular switch that activates a set of genes involved in oxygen homeostasis, energy metabolism, and angiogenesis, among other critical processes [11]. Under hypoxic conditions, HIF-1α stabilizes (i.e., it is not proteasomally degraded) and translocates to the nucleus, forming a complex with DNA and activating the gene expression necessary for adaptation to low oxygen levels [12]. Recent studies have suggested that hypoxia or low oxygen levels in the pelvis activate HIF-1α, triggering a cascade of events that enhance the expression of VEGF and other angiogenic factors such as TGF-β1 and ANG1/2, among others [13].

The second key group of promoters of angiogenesis and vascular remodeling in the endometrium—which act by regulating the growth of blood vessels, their maturation, and their regression through interaction with VEGF—are angiopoietins [14]. Four subtypes of angiopoietins (ANG1, ANG2, ANG3, and ANG4) have previously been described. Two such angiopoietins, ANG1 and 2, are deeply involved in angiogenesis [15]. ANG2 is expressed selectively in the ovary, uterus, and placenta, which are tissues that undergo significant physiological angiogenesis. Its expression is mainly localized in the glandular epithelium and endothelium of the uterus. At the same time, ANG1 is widely expressed by cells, and in the endometrium, it is mainly expressed in the stroma surrounding blood vessels in the secretory phase of the cycle [16]. The action of both ANG1 and ANG2 on vascular endothelial cells is mediated by their binding to the tyrosine kinase receptor (TIE-2) with immunoglobulin-like and EGF-like domains, in which they show similar affinity for both angiopoietins [17]. Although ANG1 and ANG2 are similar in structure, their biological activities differ significantly. ANG1 acts as a paracrine agonist on TIE-2, leading to dimerization of the receptor and inducing its phosphorylation, which then activates target signaling molecules, increases the association of endothelial cells with pericytes and vascular smooth muscle cells, and stimulates maturation of the vascular network [18].

On the contrary, ANG2 is an antagonist of ANG1. ANG2 promotes the release of the cell matrix and destabilization of the existing network of vessels. It initiates neovascularization in the presence of VEGF and, in its absence, blocks the recruitment of periendothelial supporting cells, which leads to the destabilization and regression of blood vessels [19,20]. Balance between the expression of ANG1 and ANG2, i.e., the ratio of ANGPT2 to ANGPT1 (the index of vascular instability), is essential for blood vessels’ physiological formation, development, and stabilization [21]. Vascular maturation occurs during the secretory phase of the menstrual cycle, which is regulated by progesterone. Progestins decrease ANG2 production and maintain ANG1 levels, thereby reducing the ANG2-to-ANG1 ratio [22].

The development of the vascular system in the human endometrium is controlled at the physiological and pathological level by the regulator of tissue morphogenesis, TGF-β1; it is also a potent inhibitor of the proliferation of most cell types [23]. TGF-β1 induces angiogenesis in vivo, but in vitro, it inhibits endothelial cell proliferation, migration, and proteolytic activity and reduces VEGFR2 expression [24]. In contrast to the activity of VEGF, TGF-β1 induces the apoptosis of endothelial cells because, during angiogenesis, apoptosis is required to sever the newly formed vascular network, and its inhibition leads to the formation of abnormal vessels [25]. TGF-β1 acts by binding to the ALK1 receptor (TGF-β1 receptor 1, TpRI). The expression of ALK1 is mainly limited to endothelial cells during embryogenesis and is upregulated in sites of active angiogenesis [26]. Congenital deficiency of ALK1 causes embryonic death in the gestational period and leads to defects in angiogenesis [27]. In humans, ALK1 mutations cause hereditary hemorrhagic telangiectasia, which is the absence of a capillary bed in certain vascular regions [24].

The exact signaling processes of the angiogenic pathway in the pathogenesis of endometriosis are mostly unknown. However, the crucial role of significant immune dysregulation in localized inflammation, hyperproliferation, reduced physiological apoptosis, and increased angiogenesis has been observed and described [2]. The aim of this study is to investigate the mRNA expression levels of key angiogenic factors (VEGF-A, TGF-β1, ANG1/2, and HIF-1α) in endometrial and ovarian endometrioid cell lines and elucidate their impact on cell migration, thereby advancing our understanding of the mechanisms underlying pathological angiogenesis in these conditions.

## 2. Materials and Methods

### 2.1. Cell Lines and Cultivation Protocols

We provided experiments on five epithelial cell lines, namely HME1, 12Z, A2780, RL-95-2, and SK-UT-1. The HME1 cell line (ExPASy) is an hTERT-immortalized cell line exhibiting epithelial morphology; it was isolated from the breast of a 53-year-old female patient undergoing reduction mammoplasty surgery and who had no history of breast cancer. It was then cultured in complete (with 10% fetal bovine serum (FBS), 100 U/mL penicillin, 0.1 mg/mL streptomycin, and 1.25 µg/mL amphotericin) tissue cultivation medium (human mammary epithelial cell growth medium (MEBM)) mixed with the nutrient mixture medium F-12 Ham (1:1). We used HME1 cells, a healthy mammary gland cell line representing a model of physiological angiogenesis. The epithelial tissue of the breast, as well as the endometrium of the uterus, responds to hormonal stimuli released periodically during a woman’s menstrual cycle; that is why it is a suitable replacement for the endometrial epithelial line. The 12Z cell line (a donation from Prof. Anna Starzinski-Powitz, Goethe-Universität, Frankfurt) is a SV40 virus-immortalized cell line that we obtained from a 37-year-old female patient undergoing laparoscopy. This cell line exhibits expression of markers of endometriotic lesions that can be seen in vivo. A2780 cells (a donation from Dr. Martina Šemeláková PhD., Pavol Jozef Šafárik University, Košice) form a human ovarian cancer cell line that was established from the endometrioid adenocarcinoma of an untreated patient that we cultured in complete Roswell Park Memorial Institute (RPMI) 1640 Medium. The RL-95-2 epithelial cell line was obtained from a moderately differentiated grade 2 adenosquamous carcinoma of the endometrium. This cell line contains malignant glandular and malignant squamous components. The RL-95-2 cells were cultured in complete Dulbecco’s Modified Eagle Medium combined with nutrient mixture medium (F-12 Ham) in a ratio of 1:1 with 0.005 mg/mL insulin. The SK-UT-1 cell line (a donation from Prof. Graier, Medical University of Graz) is an epithelial cell line from the uterus with a mixed mesodermal tumor containing carcinomatous and sarcomatous components. The SK-UT-1 cells were cultured in complete Dulbecco’s Modified Eagle Medium (DMEM).

All used tissue cultures were cultivated in a thermal incubator at 37 °C in a 5% CO_2_ atmosphere.

### 2.2. Wound Healing Assay

The wound healing assay is an in vitro scratch assay for assessing cell migration; it is an easy, low-cost, and well-developed method (10.1038/nprot.2007.30). For the scratch assay, the tested cells were seeded in a 6-well plate (5 × 10^5^ cells/well), and at circa 90% confluence, the cell monolayer was scraped off. After 0, 12, 24, and 48 h of incubation in complete media, an inverted microscope (Motic AE31E Series, Motic Hong Kong Limited, Hong Kong) (magnification, ×100) was used to capture the images. Migration efficiency was calculated using ImageJ.

### 2.3. mRNA Isolation and PCR

The total RNA was obtained from a cell suspension using a modified manufacturer’s protocol using an RNeasy mini kit (Qiagen; Hilden, Germany). Isolated nucleic acid was transcribed into cDNA using a ProtoScript First Strand cDNA synthesis kit (New England Biolabs; Ipswich, MA, USA) on a thermocycler (Techne TC-3000X). The qRT-PCR amplification was performed using SensiMIX II (Bioline Meridian Bioscience; London, UK) on a Rotor-Gene Q thermocycler (Qiagen; Hilden, Germany). The obtained data were analyzed with Rotor-Gene Q 2.5.3 software (Qiagen; Hilden, Germany). Relative gene expression was normalized to the housekeeping gene β-Actin (the stability of gene expression is shown in Supplementary Figure S1). The primer sequences are listed in Table 1.

### 2.4. Statistical Analysis

The presented experimental data of relative gene expression were measured in ten replicates, and data on cell migration were measured in duplicate. Data were evaluated using GraphPad Prism 8.0.1, representing the mean values ± standard deviation (SD) of ten independent measurements. An ordinary one-way ANOVA was used (Tukey’s multiple comparisons test). Statistically significant results were found to have a *p*-value < 0.05 (*), a *p*-value < 0.01 (**), and a *p*-value < 0.001 (***). The 95.00% confidence intervals (CIs) with a difference range were evaluated using GraphPad Prism 8.0.1.

## 3. Results

### 3.1. Angiogenesis Regulated via the VEGF-A/TGF-β1 Axis

**Figure 1:**
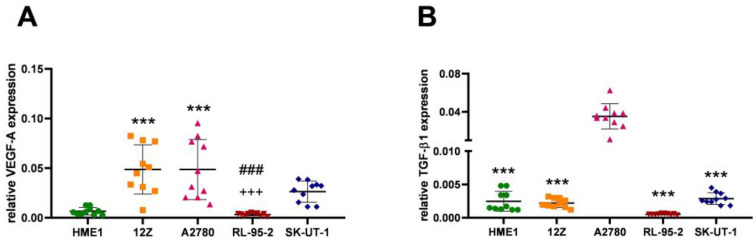
The relative gene expression of pro-angiogenic VEGF-A factor in (**A**) was determined to be significantly upregulated in cell lines 12Z (*p* < 0.0001; ***) and A2780 (*p* < 0.0001; ***) compared to the HME1 cell line with normal angiogenesis. The upregulation of VEGF-A mRNA expression in the mixed mesodermal cell line SK-UT-1 was insignificant compared to HME1. Adenosquamous RL-95-2 cells expressed non-significant downregulation of VEGF-A compared to HME1 cells. The expression of VEGF-A between 12Z and Rl-95-2 cells (*p* < 0.0001; ###) and between A2780 and RL-95-2 (*p* < 0.0001; +++) exhibited a significant change. (**B**): The expression of TGF-β1, the antagonist of VEGF-A, showed significant elevation in the A2780 (*p* < 0.0001; ***) cell line compared to the other experimental tissue models.

Angiogenesis is regulated by the action of VEGF-A in cooperation with TGF-β1. These two transcription factors act as antagonists to each other in the process of angiogenesis. The level of relative gene expression of VEGF-A (Figure 1A) in the five cell lines (HME1, a control cell line with normal angiogenesis; 12Z, with ectopic endometriosis; A2780, originating in ovarian adenoma endometriosis; and RL-95-2 and SK-UT-1, which are malignant cancerous cells) used varied based on the cells’ angiogenic properties. The cell lines 12Z (*p* < 0.0001) and A2780 (*p* < 0.0001) expressed significantly elevated VEGF-A mRNA compared to HME1. Cell line SK-UT-1 expressed a non-significant increase in VEGF-A gene expression (*p* = 0.1272), and RL-95-2 expressed a non-significant decrease in VEGF-A mRNA (*p* = 0.9940). The relative mRNA level of VEGF-A is listed in Table 2.

TGF-β1 represents apoptotic processes linked with extracellular matrix decomposition during vessel growth. Significant elevation (*p* < 0.0001 and 95.00% CI with a difference range of −0.04030 to −0.02511) of TGF-β1 expression was determined in the A2780 cells in comparison with the rest of the experimental data (Figure 1B, Table 2). This phenomenon could represent substantial angiogenic activity in A2780 cells.

**Figure 2:**
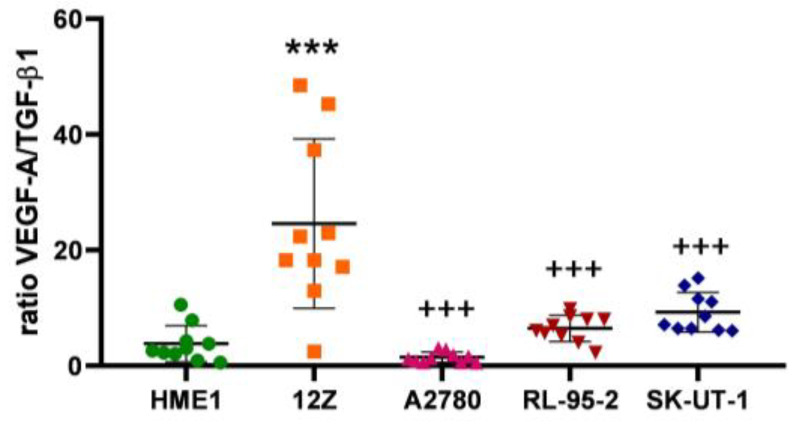
The ratio of VEGF-A to TGF-β1 increased significantly in the 12Z (*p* < 0.0001; ***) cells compared to the HME1 controls. Other considerable changes were observed in the alternative experimental tissue models (*p* < 0.0001; +++). The A2780 cell line expressed a decreased ratio of VEGF-A to TGF-β1, which may reflect the pleiotropic effect of TGF-β on angiogenic processes.

The ratio of VEGF-A to TGF-β1 indicates the pro-angiogenic activities of cells. We observed a significantly elevated ratio of VAGF-A to TGF-β1 (Figure 2) in 12Z (*p* < 0.0001) in comparison with the rest of the used cells. This could represent the strong tendency of the ectopic lesion to adhere and spread over the tissue and organs, as endometriosis is notable for the development of new vessels and nerve systems in its lesions.

### 3.2. Angiogenesis Regulated via the ANG2/ANG1 Axis

**Figure 3:**
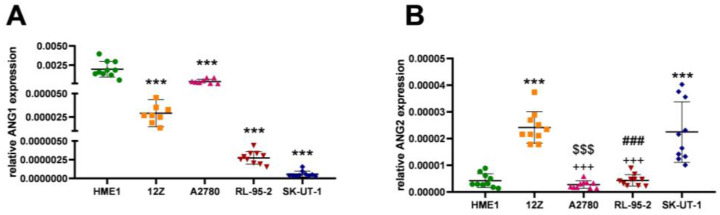
Relative gene expression in (**A**): Angiopoietin 1 was significantly elevated in the HME1 cell line (*p* < 0.0001; ***) compared to the rest of the studied cells. The endometriotic cell line 12Z and endometrioid adenocarcinoma cells A2780 showed higher mRNA levels of ANG1 compared to solid tumor cell lines RL-95-2 and SK-UT-1. (**B**): Angiopoietin 2 was expressed significantly more in the 12Z (*p* < 0.0001; ***) and SK-UT-1 (*p* < 0.0001; ***) cell lines than in HME1. The level of ANG2 in 12Z was also significantly higher than in A2780 (*p* < 0.0001; +++) and RL-95-2 (*p* < 0.0001). SK-UT-1 also expressed significantly increased ANG2 compared with A2780 (*p* < 0.0001; $$$) and RL-95-2 (*p* < 0.0001; ###).

Angiopoietins are regulators of vessel stability and spread. ANG1 promotes blood vessel maturation and stabilization, and ANG2 is responsible for vessel remodeling; ANG2 concurs with ANG1 in binding to the Tie2 receptor. During experimental analysis, HME1 cells showed significantly higher expression of ANG1 mRNA (*p* < 0.0001) compared to the rest of the cell lines (Figure 3A, Table 3), which could be explained by the formation and complete maturation of normal blood vessels. The relative gene level of ANG2 was significantly increased (*p* < 0.0001) in 12Z and SK-UT-1 cells compared to HME1 (Figure 3B). The change in ANG2 mRNA between 12Z and A2780 and RL-95-2 also showed a significant decrease (*p* < 0.0001) in cancerous cell lines. The same trend was observed with the SK-UT-1 cell line compared to A2780 (*p* < 0.0001) and RL-95-2 cells (*p* < 0.0001) (Figure 3B, Table 3).

**Figure 4:**
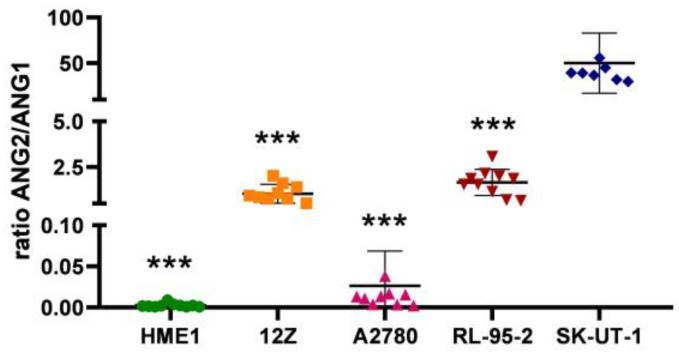
Elevation in the ratio of ANG2 to ANG1 indicates pathological angiogenesis. Cell lines SK-UT-1 showed significant elevation (*p* < 0.0001; ***) in the ratio of ANG2 to ANG1 compared to HME1, 12Z, A2780, and RL-95-2 cells.

The ratio of ANG2/ANG1 may be a helpful mortality predictor, as described in previous studies [28,29,30,31,32]. If the ratio favors ANG2 expression, it could result in poor clinical outcomes. The ratio of ANG2 to ANG1 was significantly higher in SK-UT-1 cells compared to the rest of the cell lines (*p* < 0.0001) (Figure 4). The ANG2/ANG1 ratio expressed non-significant elevation in 12Z and RL-95-2 cells compared to HME1. The A2780 cell line showed a decrease in the ratio of ANG2 to ANG1 compared to the rest of the pathological cell lines (Figure 4), suggesting different angiogenic axis activation in this pathological microenvironment.

### 3.3. Angiogenesis Regulated via Hypoxia

**Figure 5:**
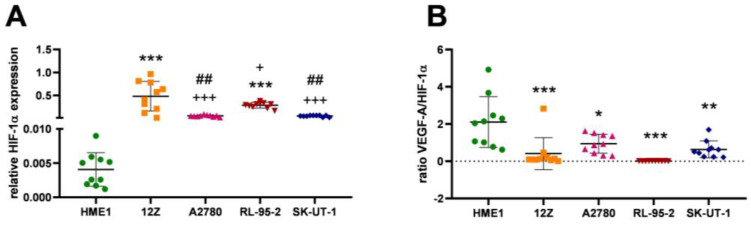
Hypoxia-inducible transcription factor 1α is known to be elevated in pathological conditions. (**A**): The expression of HIF-1α was significantly elevated in 12Z (*p* < 0.0001; ***) and RL-95-2 (*p* = 0.0009; ***) compared to HME1 cells. A significant change was also observed between 12Z vs. A2780 (*p* < 0.0001; +++), 12Z vs. RL-95-2 (*p* = 0.0326; +), and 12Z vs. SK-UT-1 (*p* < 0.0001; ***). The HIF-1α expression between A2780 vs. RL-95-2 (*p* = 0.0085; ##) and RL-95-2 vs. SK-UT-1 (*p* = 0.0072; ##) also changed significantly. (**B**): The ratio of HIF-1α/VEGF-A is significantly elevated in pathological conditions of 12Z (*p* = 0.0002; ***), A2780 (*p* = 0.0149; *), RL-95-2 (*p* < 0.0001; ***), and SK-UT-1 (*p* = 0.0012; **) cells compared to HME1 controls.

Hypoxia is the primary stimulus for the formation of a new blood supply to compensate for the needs of cells. The mimicking of hypoxia is also a feature of pathological metabolism that serves to expel the apoptotic stimuli and ensure survival in pathological conditions. Figure 5A and Table 4 indicate the significant elevation of HIF-1α in 12Z (*p* < 0.0001) and RL-95-2 (*p* = 0.0009) compared to HME1. The elevation in HIF-1α expression in A2780 and SK-UT-1 was non-significant compared with HME1. The 12Z cell line expressed a significant increase in HIF-1α compared to A2780 (*p* < 0.0001), RL-95-2 (*p* = 0.0326), and SK-UT-1 (*p* < 0.0001). The gene expression of HIF-1α in RL-95-2 cells underwent a significant increase compared with A2780 (*p* = 0.0085) and SK-UT-1 (*p* = 0.0072) cells.

The VEGF-A/HIF-1α axis regulates tumor progression [33], as the hypoxic upregulation of VEGF-A is required to promote the angiogenic phenotype. The favorable ratio of VEGF-A/HIF-1α to VEGF-A could represent physiological angiogenesis in dominantly normoxic conditions. If the ratio of VEGF-A to HIF-1α shifts to HIF-1α, metabolic adaptation in molecular subtypes of the cancer cells [34] and the pathological formation of blood vessels in the hypoxic microenvironment may be indicated. The ratio of VEGF-A to HIF-1α was significantly elevated in HME1 compared to 12Z (*p* = 0.0002), to A2780 (*p* = 0.0149; 95.00% CI with a difference range of 0.1657 to 2.159), to RL-95-2 (*p* < 00001; 95.00% CI with a difference range of 1.095 to 3.089), and also to SK-UT-1 (*p* = 0.0012) (Figure 5B). 

### 3.4. Wound Healing Assay

**Figure 6:**
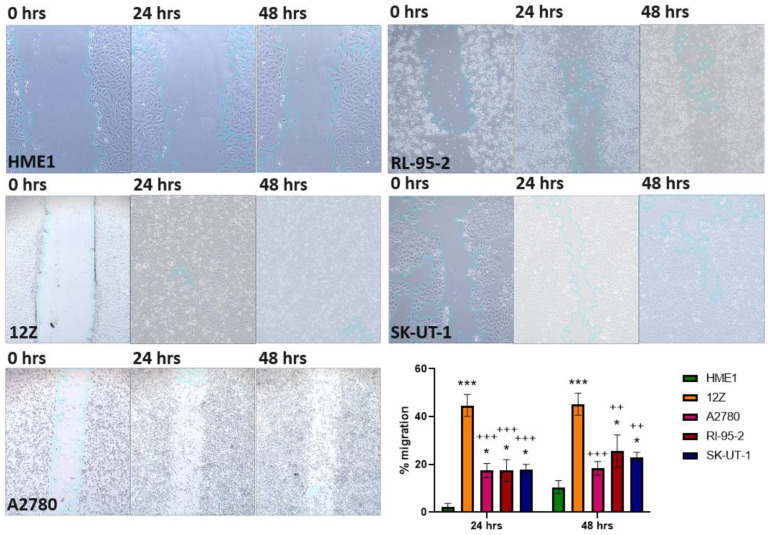
Cell migration determination by wound healing assay. The assays were performed at 0, 24, and 48 h in HME1, 12Z, A2780, RL-95-2, and SK-UT-1 cells. The bar plot represents the quantification of % of cell migration as determined by the rate of HEM1, 12Z, A2780, RL-95-2, and SK-UT-1 cells moving towards the scratched area at a given time (24 and 48 h). The bar plot shows a significant change in cell migration within 24 h in HME1 vs. 12Z (*p* < 0.0001; ***), HME1 vs. A2780 (*p* < 0.0001; ***), HME1 vs. RL-95-2 (*p* = 0.0166; *), HME1 vs. SK-UT-1 (*p* = 0.0151; *), 12Z vs. A2780 (*p* = 0.0002; +++), 12Z vs. RL-95-2 (*p* = 0.0002; +++), and 12Z vs. SK-UT-1 (*p* = 0.0003; +++). Also shown is the significant change in cell migration within 48 h in HME1 vs. 12Z (*p* < 0.0001; ***), HME1 vs. RL-95-2 (*p* = 0.0175; *), HME1 vs. SK-UT-1 (*p* = 0.0485; *), 12Z vs. A2780 (*p* = 0.0003; +++), 12Z vs. RL-95-2 (*p* = 0.003; ++), and 12Z vs. SK-UT-1 (*p* = 0.0012; ++).

To analyze angiogenic properties based on in vitro cell migration, a wound healing assay (Figure 6) was performed on the control cell line with normal angiogenesis, HME1; the ectopic endometriosis cell line, 12Z; endometrioid adenocarcinoma, A2780; and malignant tumor cells of uterine endometrium, RL-95-2 and SK-UT-1. The cell migration of control HME1 was, as expected, at the lowest level at both analyzed time points (24 h = 2.226% and 48 h = 10.468%). The highest cell migration was determined in endometriotic 12Z (24 h = 44.682% and 48 h = 45.237%). The observed percentages of cell migration in the other cell lines are shown in Table 5.

## 4. Discussion

Despite existing knowledge of the importance of angiogenesis in the pathogenesis of uterine diseases, the precise role of angiogenic factors remains a subject of ongoing scientific research. It is well documented that molecular-level changes manifest themselves significantly earlier than phenotypic changes. Therefore, understanding the cooperation of the angiogenic markers VEGF-A, TGF-β1, ANG1, ANG2, and HIF-1α can facilitate their early diagnosis or help in the choice of an appropriate therapeutic approach. In the present study, a cell line (HME1) of healthy epithelial tissue from the mammary gland was selected as a model of normal angiogenesis; this is because the ratios of VEGF-A to TGF-β1 and ANG2 to ANG1 alongside the expression of HIF-1α are physiological metrics that signal normal blood vessel formation without pathological destabilization of the vascular barrier [35,36].

Vascular endothelial factors are one of the most important transcription factors that regulate angiogenesis. Increased gene and protein expression of VEGF-A has been described in peritoneal fluid [37], ectopic endometriotic lesions [38], blood serum [32,39], and in vitro/in vivo conditions. VEGF-A is a key angiogenic factor not only in uterine pathologies but also in other diseases, including malignant transformations and chronic inflammatory, infectious, autoimmune, or metabolomic diseases, as has been well documented [3,40]. Increased expression of VEGF-A in ectopic tissue, as described by Arablou et al. [41], correlates with the results presented in this study (Figure 1A—12Z). Similarly, Xia et al. [42] found the expression of VEGF-A to be increased in ovarian carcinoma (Figure 1A—A2780) cells compared to normal ovarian epithelial cells. The elevated expression of VEGF-A has been proven to contribute to a more aggressive phenotype of the disease [43]. As a result of the progression of cancer, VEGF-A expression may decrease (Figure 1A—RL-95-2), but the nature of this process has not been elucidated [44]. This regression may be caused by different metabolic processes in a mixed mesodermal tumor, where cells express this transcription factor differently, i.e., carcinoma cells show higher expression of VEGF-A than sarcoma cells [45]. However, the majority of neoplastic cells show a higher expression of VEGF-A (Figure 1A—SK-UT-1) [46].

In addition to the increased expression of VEGF-A, angiogenesis also requires the cooperation of TGF-β1, which has a different effect on endothelial cells. VEGF-A can protect against endothelial cell apoptosis; TGF-β, on the contrary, induces apoptosis [24]. Apoptosis and the breakdown of the intercellular mass are crucial for the severing of tissue by new vessels. The most critical factor in vascular morphogenesis is the pleiotropic effect of TGF-β factors [47], as there are conflicting data on the level of TGF-β within uterine pathologies [48,49]. TGF-β’s pleiotropic effect, taking into account the impact of progesterone on TGF-β signaling (which effectively suppresses the TGF-β signaling pathway in malignant cells), may therefore be closely related to the sensitivity of cells to individual hormonal stimuli [50]. The increased expression of TGF-β factors (including TGF-β receptors) is demonstrably increased in recurrent mixed carcinomas compared to non-recurrence [51]. Tumor aggressiveness is mediated by TGF-β’s induction of Smad 2/3 [52]. In tumor tissues, the expression of TGF-β is reduced (Figure 1B—RL-95-2) and is responsible for the incomplete maturation of the vascular wall, which leads to its rupture [53]. TGF-β increases the expression of VEGF-A at both the gene and protein levels [54]. A decrease in the ratio of VEGF-A to TGF-β suggests that VEGF-A production under hypoxic conditions may be independent of TGF-β-mediated signaling [55].

Hypoxia is a frequent concomitant of pathological diseases, which leads to the activation of hypoxia-induced factors. Hypoxia-induced transcription factor 1α has been described in increased expression for many pathologies, including gynecological. This increase in the level of HIF-1α is a measure of invasiveness and metastasis (Figure 5A—12Z, A2780, RL-95-2, and SK-UT-1) [56,57], which is mediated through the orientation of energy metabolism almost exclusively by the process of glycolysis and the increase in angiogenic factors; this leads to the prompt formation of a vascular network and thus to a rapid exchange of metabolites between pathological cells [58]. Hypoxia deepens the influence of VEGF-A (as the downstream target of HIF-1α’s action is VEGF-A). Thus, an increase in the ratio of HIF-1α/VEGF-A (Figure 5B) is responsible for a more aggressive phenotype of uterine disease [59].

Another primary mode of angiogenic regulation is predominantly through angiopoietins 1 and 2, which compete to bind to the Tie2 receptor. ANG2 has a higher affinity to Tie2 and, in an excessive concentration, suppresses the effect of ANG1 on the formation of a blood network [60]. The ANG2-to-ANG1 ratio increases in the early stages of angiogenesis [61], as was observed in the 12Z cell line (Figure 3 and Figure 4). With the progression of disease and changes in the demands that cells make of the blood supply, this ratio can shift. The research of Yamamoto et al. aimed to monitor the activity of angiopoietins and described their varied expression in ovarian tumors, cysts, and endometriosis [62]. Hu and Cheng described an increase in ANG2 and a decrease in ANG1 (i.e., an overall decrease in the ANG1 to ANG2 ratio) during the simultaneous elevation of VEGF-A, as described in the results of VEGF-A expression and the ratio of ANG2 to ANG1 in Figure 1A and Figure 4. This event increased microvessel density in pathological ovarian tissue [63].

The increased expression of ANG2 in inflammatory hypoxic conditions stabilizes the ANG2/Tie2 axis. It thus inhibits the stabilizing effect of ANG1, causing the destabilizing impact of ANG2 to increase. Thereafter, the proliferative and migratory effect of VEGF-A leads to vessel growth and pathological angiogenesis [43]. Destabilized newly formed vessels burst easily, have an atypical structure, and are incapable of complete function, leading to an increase in angiogenic factors in an attempt to compensate for the non-physiological vascular network, thereby exacerbating pathological angiogenesis [64].

Endothelial cell migration is essential to angiogenesis within various physiological and pathological processes [65]. This tightly regulated cell migration process is VEGF-dependent [39,66], as also confirmed by increased cell migration (Figure 6) in the 12Z, A2780, and SK-UT-1 cells in our study, all of which demonstrated higher VEGF-A expression (Figure 2). The cell migration of A2780 cells was also significantly elevated because TGF-β1 induces cell motility and is crucial to cancer cells’ malignant phenotype [67]. 

## 5. Conclusions

The pivotal role of angiogenic factors in uterine pathogenesis is indisputable. There is substantial evidence of their importance both in resistance to treatment and worse disease outcomes. Uterine pathology is more common now than ever before. Even though scientists are familiar with the critical pro- and anti-angiogenic factors, the expression of those markers can differ based on the type and stage of the disorder. This article lists several angiogenic disorders of two different origins (endometriosis, ovarian endometrioid adenocarcinoma, and endometrial carcinoma) and compares them with a normal angiogenic cell line. The VEGF-A/TGF-β1, ANG2/ANG1, and VEGF-A/HIF-1α axes are critical for pathological angiogenesis. The tested tissue cell culture of endometriosis (12Z) expressed a high ratio of VEGF-A to TGF-β1, ANG2 to ANG1, and VEGF-A to HIF-1α, which could explain the invasiveness and high migration potential of endometriosis observed in vivo. The endometrioid adenocarcinoma (A2780) cell line expressed a high level of TGF-β1 that led to a low ratio of VEGF-A to TGF-β1, which could be explained by the excessive decomposition of extracellular mass. Moderately differentiated adenosquamous carcinoma (RL-95-2) cells did not significantly change VEGF-A or TGF-β1 expression when compared with a control cell line exhibiting normal angiogenesis (HME1). High migration potential and invasivity appear to be the consequence of a high ANG2/ANG1 ratio. Finally, mixed mesodermal tumor (SK-UT-1) cells expressed an enormous ANG2 to ANG1 ratio, making cells more invasive. In all pathological conditions, HIF-1α was significantly elevated, which also supports theories regarding the invasiveness of cells with high angiogenic potential. We are aware of the heterogeneity of endometriosis as well as endometrial cancer, which is also reflected in the variability in angiogenesis between the tested cells. A similar phenomenon can be expected between patients, as well as individual stages of the disease between patients as individuals or in groups, which limits broad conclusions from the presented data. The recognition of the driven angiogenic axis is crucial for appropriate pre-screening tests.

## Figures and Tables

**Table 1:** mRNA primer sequences used to assess relative gene expression using qRT-PCR.

Target Gene	Forward	Reverse
Β-Actin	AGAGCCCAGTCTTCATTGCT	TGTCCTGTTGCATACCGTCT
VEGF-A	ATAAGTCCTGGAGCGTTCCCT	GTTTAACTCAAGCTGCCTCGC
TGF-β1	AAGATGGAGAGAGGACTGCG	AGAGGGAGAGAGAGGGAGTG
ANG1	CGTGGAACCGGATTTCTCTTC	TGGGCCATCTCCGACTTCAT
ANG2	AACCAAACAGCGGAGCAAAC	AGGGAGTGTTCCAAGAGCTG
HIF-1α	GACCGATTCACCATGGAGGG	GTGGCAACTGATGAGCAAGC

**Table 2:** The average relative gene expression ± SD of angiogenic factors VEGF-A and TGF-β1 in the experimental cell line of uterine pathologies.

Cell Line	VEGF-A	TGF-β1
HME1	0.006709 ± 0.003730	0.002470 ± 0.001492
12Z	0.048710 ± 0.024825	0.002206 ± 0.000678
A2780	0.048700 ± 0.030207	0.035170 ± 0.013240
RL-95-2	0.003395 ± 0.001363	0.000519 ± 0.000094
SK-UT-1	0.026450 ± 0.010603	0.002874 ± 0.000883
	95.00% CI VEGF-A	95.00% CI TGF-β1
HME1 vs. 12Z	−0.06514 to −0.01887	−0.007337 to 0.007860
HME1 vs. A2780	−0.06512 to −0.01886	−0.04030 to −0.02511
HME1 vs. RL-95-2	−0.01982 to 0.02645	−0.005648 to 0.009549
HME1 vs. SK-UT-1	−0.04288 to 0.003388	−0.008005 to 0.007192
12Z vs. A2780	−0.02312 to 0.02315	−0.04057 to −0.02537
12Z vs. RL-95-2	0.02219 to 0.06845	−0.005909 to 0.009288
12Z vs. SK-UT-1	−0.0008711 to 0.04539	−0.008267 to 0.006930
A2780 vs. RL-95-2	0.02217 to 0.06844	0.02706 to 0.04225
A2780 vs. SK-UT-1	−0.0008844 to 0.04538	0.02470 to 0.03990
RL-95-2 vs. SK-UT-1	−0.04619 to 7.441 × 10^−5^	−0.009956 to 0.005241

**Table 3:** The average relative gene expression ± SD of angiogenic factors ANG1 and ANG2 in experimental cell lines of uterine pathologies.

Cell Line	ANG1	ANG2
HME1	0.001974 ± 0.001028	0.000004 ± 0.000003
12Z	0.000029 ± 0.000015	0.000024 ± 0.000006
A2780	0.000035 ± 0.000297	0.000003 ± 0.000001
RL-95-2	0.000003 ± 0.000001	0.000004 ± 0.000002
SK-UT-1	0.000001 ± 0.000004	0.000022 ± 0.000011
	95.00% CI ANG1	95.00% CI ANG2
HME1 vs. 12Z	0.001337 to 0.002553	−2.750 × 10^−5^ to −1.238 × 10^−5^
HME1 vs. A2780	0.001014 to 0.002231	−6.098 × 10^−6^ to 9.020 × 10^−6^
HME1 vs. RL-95-2	0.001363 to 0.002580	−7.684 × 10^−6^ to 7.434 × 10^−6^
HME1 vs. SK-UT-1	0.001365 to 0.002582	−2.577 × 10^−5^ to −1.065 × 10^−5^
12Z vs. A2780	−0.0009312 to 0.0002856	1.384 × 10^−5^ to 2.896 × 10^−5^
12Z vs. RL-95-2	−0.0005822 to 0.0006346	1.226 × 10^−5^ to 2.738 × 10^−5^
12Z vs. SK-UT-1	−0.0005801 to 0.0006367	−5.829 × 10^−6^ to 9.289 × 10^−6^
A2780 vs. RL-95-2	−0.0002594 to 0.0009574	−9.145 × 10^−6^ to 5.973 × 10^−6^
A2780 vs. SK-UT-1	−0.0002573 to 0.0009595	−2.723 × 10^−5^ to −1.211 × 10^−5^
RL-95-2 vs. SK-UT-1	−0.0006062 to 0.0006106	−2.565 × 10^−5^ to −1.053 × 10^−5^

**Table 4:** Average relative gene expression ± SD of hypoxia-inducible transcription factor 1α in the experimental cell lines of uterine pathologies.

Cell Line	HIF-1α
HME1	0.004057 ± 0.002464
12Z	0.481800 ± 0.320725
A2780	0.053650 ± 0.018822
RL-95-2	0.284200 ± 0.061784
SK-UT-1	0.049860 ± 0.016882
	95.00% CI HIF-1α
HME1 vs. 12Z	−0.6639 to −0.2915
HME1 vs. A2780	−0.2358 to 0.1366
HME1 vs. RL-95-2	−0.4663 to −0.09396
HME1 vs. SK-UT-1	−0.2320 to 0.1404
12Z vs. A2780	0.2419 to 0.6143
12Z vs. RL-95-2	0.01139 to 0.3837
12Z vs. SK-UT-1	0.2457 to 0.6181
A2780 vs. RL-95-2	−0.4167 to −0.04437
A2780 vs. SK-UT-1	−0.1824 to 0.1900
RL-95-2 vs. SK-UT-1	0.04816 to 0.4205

**Table 5:** Percentage ± SD cell migration at 24 h and 48 h after scratch.

Cell Line	24 h	48 h
HME1	2.226 ± 1.237	10.468 ± 2.254
12Z	44.682 ± 23.244	45.237 ± 17.074
A2780	17.464 ± 3.772	18.427 ± 3.746
RL-95-2	17.468 ± 16.385	25.578 ± 16.192
SK-UT-1	17.712 ± 2.431	23.083 ± 2.319

## Data Availability

The datasets used and/or analyzed during the current study are available from the corresponding author upon reasonable request.

## Supplementary Materials

The following supporting information can be downloaded at https://www.mdpi.com/article/10.3390/cancers16162772/s1, Figure S1: The Ct values of two tested reference gene—β-Actin and GAPDH.
