# Supplementary material for: The Pivotal Role of the Key Angiogenic Factors in the Development of Endometrioid Pathologies of the Uterus and Ovary
Source: Cancers (Basel). 2024 Aug 6;16(16):2772. doi: 10.3390/cancers16162772 (PMC11352877; doi:10.3390/cancers16162772)
Supplement: Supplementary file 1 [file cancers-16-02772-s001.zip › cancers-3084219-supplementary.pdf]

cancers

Article

The Pivotal Role of the Key Angiogenic Factors in the Development of Endometrioid Pathologies of the Uterus and Ovary

Gabriela Sabolová [1], Ivana Špaková [1,*], Peter Artimovič [1], Peter Bohuš [2], Miroslava Rabajdová [1] and Mária Mareková [1]

[1] Department of Medical and Clinical Biochemistry, P. J. Šafárik University in Košice, Trieda SNP 1, SK-04011 Košice, Slovakia; gabriela.sabolova@student.upjs.sk (G.S.); peter.artimovic@student.upjs.sk (P.A.); miroslava.rabajdova@upjs.sk (M.R.); maria.marekova@upjs.sk (M.M.)

[2] Department of Pathology, P. J. Šafárik University in Košice, Trieda SNP 1, SK-04011 Košice, Slovakia; peter.bohus@student.upjs.sk (P.B.)

* Correspondence: ivana.spakova@upjs.sk; Tel.: +421-55-234-3259

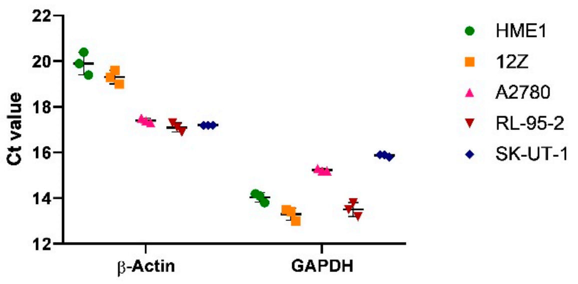

Figure S1. The Ct values of two tested reference genes – β-Actin and GAPDH.
